# Historical path of paraphrenia

**DOI:** 10.1192/j.eurpsy.2021.1385

**Published:** 2021-08-13

**Authors:** A. Hernández Mata, A. Sotillos Gómez, P. Marco Coscujuela, B. Ordóñez Méndez

**Affiliations:** Psychiatry, Hospital Universitario de Getafe, Getafe, Spain

**Keywords:** Paraphrenia

## Abstract

**Introduction:**

Paraphrenia is a psychotic disorder characterized by an insidious development of a vivid and exuberant delusional system, accompanied by hallucinations and confabulations, without a personality deterioration. It is considered to be an intermediate entity between the disorganization of schizophrenia and the systematization of a delusional disorder.

**Objectives:**

Develop knowledge about paraphrenia as an individualized diagnostic entity and its historical path through the classical authors’ texts.

**Methods:**

Extensive research on the historical path of the paraphrenia diagnostic entity was carried out, as well as the current situation of the term.

**Results:**

In the German psychiatry it was Karl Kahlbaum who first introduced the term of paraphrenia. Later many authors of the German psychiatry delved into this diagnostic entity. Emil Kraepelin described four different subtypes of paraphrenia: paraphrenia systematica, expansiva, confabulans and phantastica. However, other authors such as Kleist or Bleuler, considered paraphrenia should not be judge as an individualized diagnostic entity as it should be considered inside schizophrenia, so the term disappeared in the German psychiatry. In the French psychiatry, unlike the German, the independence of chronic psychosis from schizophrenias was recognized, so the term had a longer path. Henry Ey recognized four important clinical features in this disorder: paralogical thought dominance, megalomania, confabulation and integrity of relation with reality.

**Conclusions:**

Currently the term paraphrenia is no longer considered an individualized diagnostic entity. In fact, in today’s textbooks of psychiatry paraphrenia is considered a psychotic disorder that has nothing in common with the one described by the classical authors, and it is part of the late-onset psychosis.

